# Flow condensation heat transfer coefficient and pressure drop data for R134a alternative refrigerants R513A and R450A in a 0.95-mm-diameter minichannel

**DOI:** 10.1016/j.dib.2022.108577

**Published:** 2022-09-05

**Authors:** Jordan A. Morrow, Melanie M. Derby

**Affiliations:** Kansas State University, Alan Levin Department of Mechanical and Nuclear Engineering, 3002 Rathbone Hall, 1701B Platt St, Manhattan, KS 66506, USA

**Keywords:** Low global warming potential refrigerant, heat transfer coefficient, Flow condensation, Pressure drop

## Abstract

Flow condensation heat transfer coefficients and pressure drop data were collected in a small-scale vapor compression cycle. The test section consisted of seven parallel, 0.95-mm-diameter square channels which utilized a heat flux block to measure the temperature gradients of the heat leaving during the condensation process. Heat transfer coefficients and pressure drops were measured using the temperatures and pressures measured during experiments. Experimental flow condensation heat transfer coefficient and pressure drop data are tabulated for R134a, R513A, and R450A for a range of mass fluxes (i.e., 200 – 500 kg/m^2^s) and qualities (i.e., 0.2 – 0.8) at a saturation temperature of 40°C. The heat transfer coefficient uncertainties for all experiments were ± 6.3 – 21.2%, with an average uncertainty of ± 9.8%. Data include refrigerant saturation temperature, wall temperature, mass flux, quality, condensation heat transfer coefficient and its uncertainty, and pressure drop. The data tabulated are the raw data from the paper “Flow condensation heat transfer and pressure drop performance of R134a alternative refrigerants R513A and R450A in a 0.95-mm-diameter minichannel,” published by the International Journal of Heat and Mass Transfer [1].

**Specifications Table**

**Table:** 

Subject	Mechanical Engineering
Specific subject area	Low global warming potential refrigerant heat transfer and pressure drop performance
Type of data	Table
How the data were acquired	Data were collected using a small-scale vapor compression cycle. The test section was seven parallel, 0.95-mm-diameter square channels which utilized a heat flux block to measure the temperature gradients of the heat removed during the condensation process. Heat transfer coefficients and pressure drops were measured using the temperatures and pressures measured during experiments. Temperatures were measured using Type T thermocouples (Omega TMQSS-116U-6); refrigerant pressures were measured using 0-300 psi absolute pressure transducers (Omega PX309-300A5V) and pressure drop was measured using a differential pressure transducer (Setra 2301030PD2F2DA). The refrigerant mass flow rate was measured using a rotameter (Omega FL-4SB-40C-40ST-39ST-39G-PTFE) and calibrated using a Coriolis flow meter (Micro Motion 5700). The temperatures and pressures were measured using a National Instruments data acquisition system and LabVIEW. Water flow rates were measured using a Coriolis mass flow meter (Micro Motion 5700) and collected using ProLink III software. The cycle was run until reaching steady state conditions, then the data were collected for five minutes.
Data format	Raw Analysed
Description of data collection	Experimental flow condensation heat transfer coefficient and pressure drop data are tabulated for R134a, R513A, and R450A in seven parallel 0.95-mm diameter mini-channels for a range of mass fluxes (i.e., 200–500 kg/m^2^s) and qualities (i.e., 0.2–0.8) at a saturation temperature of 40°C. The heat transfer coefficient uncertainties for all experiments were ± 6.3–21.2%, with an average of ± 9.8%.
Data source location	Kansas State University Manhattan, KS 66506 United States of America
Data accessibility	Repository name: Mendeley Data Data identification number: 10.17632/7rksfp2bbk.2 Direct URL to data: https://data.mendeley.com/datasets/7rksfp2bbk/2
Related research article	J.A. Morrow, M.M. Derby, Flow condensation heat transfer and pressure drop performance of R134a alternative refrigerants R513A and R450A in a 0.95-mm-diameter minichannel, International Journal of Heat and Mass Transfer 192 (2022) 122894.

## Value of the Data

• Flow condensation heat transfer coefficients and pressure drops of novel low global warming potential refrigerants are important for characterizing and comparing the performance of these refrigerants, as well as system design for low GWP fluids. The data in brief includes wall temperatures, which are not plotted in the article. Wall temperatures are necessary for calculating many frequently used correlations, including Thome et al. [2], Cavallini et al. [3], and Dobson and Chato [4]. While most of the data are presented in graphs in the published article, the tabulated values also benefit correlation development, improving the accuracy of low GWP refrigerant correlations.
• Design and modelling engineers will benefit from the fundamental heat transfer data comparing refrigerant performance.
• Data can be used for comparing refrigerant performance, for correlation development, and for refrigeration system modelling.

## 1 Data Description

The Mendeley dataset contains refrigerant saturation temperature, wall temperature, mass flux, quality, heat transfer coefficient, heat transfer coefficient uncertainty, and pressure drop data for R134a, R513A, and R450A in 0.95-mm-diameter minichannels. There are two data files on Mendeley Data. The first data file is Flow condensation data.pdf, which included the raw saturation temperature and pressure drop, and calculated wall temperatures, mass fluxes, qualities, heat transfer coefficients and resulting uncertainties. The second data file is Flow condensation data.xls, which contains the raw saturation temperature and pressure drop, and calculated wall temperatures, mass fluxes, qualities, heat transfer coefficients and resulting uncertainties in an Excel spreadsheet, and includes the pressure drop in kPa.

## 2 Experimental Design, Materials and Methods

Data were collected using a small-scale vapor compression cycle. The test section was seven parallel, 0.95-mm-diameter square channels which utilized a heat flux block to measure the temperature gradients of the heat removed during the condensation process. Heat transfer coefficients and pressure drops were measured using the temperatures and pressures measured during experiments. Temperatures were measured using Type T thermocouples (Omega TMQSS-116U-6); refrigerant pressures were measured using 0-300 psi absolute pressure transducers (Omega PX309-300A5V) and pressure drop was measured using a differential pressure transducer (Setra 2301030PD2F2DA). The refrigerant mass flow rate was measured using a rotameter (Omega FL-4SB-40C-40ST-39ST-39G-PTFE) and calibrated using a Coriolis flow meter (Micro Motion 5700). The temperatures and pressures were measured using a National Instruments data acquisition system and LabVIEW. Water flow rates were measured using a Coriolis mass flow meter (Micro Motion 5700) and collected using ProLink III software. The cycle was run until reaching steady state, then the data were collected for five minutes. The condensation heat transfer coefficient, *h*, is calculated,

(1) $$h=\frac{q_{ch}^{\prime \prime }}{T_{fluid}-T_{wall}}$$

where $q_{ch}^{\prime \prime }$ is the heat flux in the channel, $T_{fluid}$ is the fluid temperature, and $T_{wall}$ is the extrapolated wall temperature. The fluid temperature of two-phase condensation is calculated as the saturation temperature for azeotropic fluids (i.e., R134a, and R513A) and the bubble point temperature for zeotropic fluids (i.e., R450A). The wall temperature is measured using a thermocouple three millimeters below the channel, and extrapolated to the surface of the channel using the temperature gradient. The heat flux in the channel is calculated from the energy balance between the heat leaving the test section channel segment and the corresponding block segment:

(2) $$q_{ch}^{\prime \prime }A_{s,ch}=q_{block}^{\prime \prime }A_{block}$$

where $A_{s,ch}$ is the surface area of the test section channel's segment. The thermocouple measuring the wall temperature is 3 mm below the surface, so the wall temperature is extrapolated using the wall thermocouple and the five thermocouples used to calculate the heat flux in the block. The heat flux through each block is calculated using Fourier's Law,

(3) $$q_{block}^{\prime \prime }=-k_{cu}\frac{dT}{dy}$$

where $k_{cu}$ is the thermal conductivity of copper and $\frac{dT}{dy}$ is the temperature gradient in the vertical *y-*axis of each segment. The temperature gradient is measured for each segment using a linear regression of the form,

(4) $$\frac{dT}{dy}=\frac{\sum^{\left(y_{i}-\bar{y}\right)}\left(T_{i}-\bar{T}\right)}{\sum^{\left(y_{i}-\bar{y}\right)}{}^{2}}$$

where $y_{i}$ is the distance in the y-axis vertically down of the *i*th thermocouple from the top of the heat flux block, $T_{i}$ is the measured temperature of the *i*th thermocouple, $\bar{y}$ is the average distance in the y-axis vertically down, and $\bar{T}$ is the average measured temperature of the segment.

## Ethics Statement

This manuscript adheres to the Ethics in publishing standards.

## CRediT authorship contribution statement

**Jordan A. Morrow:** Investigation, Validation, Writing – original draft. **Melanie M. Derby:** Conceptualization, Methodology, Writing – review & editing.

## Declaration of Competing Interest

The authors declare that they have no known competing financial interests or personal relationships that could have appeared to influence the work reported in this paper.

The authors declare the following financial interests/personal relationships which may be considered as potential competing interests:

## Data Availability

Flow condensation heat transfer coefficient and pressure drop data for R134a alternative refrigerants R513A and R450A in a 0.95-mm-diameter minichannel (Original data) (Mendeley Data).

## References

[1] Morrow J.A., Derby M.M. (2022). Flow condensation heat transfer and pressure drop of R134a alternative refrigerants R513A and R450A in 0.95-mm diameter minichannels. Int. J. Heat Mass Transfer.

[2] Thome J.R., El Hajal J., Cavallini A. (2003). Condensation in horizontal tubes, part 2: new heat transfer model based on flow regimes. Int. J. Heat Mass Transfer.

[3] Cavallini A., Col D.D., Doretti L., Matkovic M., Rossetto L., Zilio C., Censi G. (2006). Condensation in horizontal smooth tubes: A new heat transfer model for heat exchanger design. Heat Transfer Eng..

[4] Dobson M.K., Chato J.C. (1998). Condensation in smooth horizontal tubes. J. Heat Transfer.

